# A Curious Case of Hypervitaminosis D

**DOI:** 10.7759/cureus.8515

**Published:** 2020-06-08

**Authors:** Neha Sharma, Eric Landsberg, Vishnu Kumar, Harvir Singh S Gambhir

**Affiliations:** 1 Internal Medicine, Maimonides Medical Center, New York, USA; 2 Internal Medicine, State University of New York (SUNY) Upstate Medical University, Syracuse, USA

**Keywords:** vitamin d, 25(oh)d, toxicity, awareness, hypervitaminosis, vitamin d toxicity, acute kidney injury, megadoses, self medication

## Abstract

The growing awareness of the beneficial health effects of vitamin D supplementation, the commonality of vitamin D deficiency, and the over-the-counter availability of vitamin D supplements has resulted in a renewed interest in vitamin D supplementation among patients as well as health care providers. We present a case of vitamin D toxicity (VDT) in a patient following self-medication with mega doses that far exceeded the prescribed dosage. He presented to us with acute kidney injury (AKI) and elevated serum 25(OH)-vitamin D levels. He was treated with intravenous hydration, loop diuretics, and prednisone and had a clinical and biochemical improvement as reflected in his labs. We suggest that physicians and health care providers be made aware of this commonly overlooked side-effect of vitamin D supplementation and the importance of regular blood levels to titrate the dose accordingly. At the same time, it is important that the public is made aware of the ill-effects of self-medicating on large doses of vitamins.

## Introduction

Vitamin D is an essential prohormone in calcium homeostasis and bone mineral metabolism [1]. It is also known to play a vital role in parathyroid hormone regulation, cell differentiation, and protection against many auto-immune diseases and cancer [2]. A deficiency of vitamin D leads to rickets and osteomalacia, where bones start losing calcium because of the negative calcium balance in the body. However, in extremely rare cases, over-correction of vitamin D can lead to hypervitaminosis D and subsequent vitamin D toxicity (VDT). Vitamin D has a wide therapeutic index, therefore, for the symptoms of toxicity to appear, the patient must take mega-doses over a long period of time. Here, we present a unique case of VDT following misinformed self-medication, in the background of chronic renal failure.

## Case presentation

A 73-year-old male with a past medical history of heart failure with reduced ejection fraction, stage-III chronic kidney disease, automatic implantable cardioverter-defibrillator in place, hypertension, atrial fibrillation not on anticoagulation, and stable myelodysplastic syndrome not on chemotherapy, presented from home with a five-day history of bilateral lower extremity pain, increased shortness of breath, and insomnia. The patient endorsed decreased oral intake and urine output but denied gross hematuria, flank pain, or costovertebral angle pain. The patient was on the following oral home medications at presentation: B complex-biotin-FA (B-complex), coenzyme Q10 (COQ-10), finasteride (Proscar) 5 mg, flaxseed oil capsules 1000 mg, furosemide (Lasix) 20 mg, hydralazine (Apresoline) 10 mg, ipratropium-albuterol (Duoneb) 0.5-2.5 mg/3 ml solution, isosorbide mononitrate (Imdur) 30 mg, magnesium citrate solution, metolazone (Zaroxolyn) 5 mg, metoprolol (Toprol-XL) 100 mg, polyethylene glycol powder (Glycolax), potassium chloride (K-Dur, Klor-con) 20 mEq, turmeric, zolpidem (Ambien) 10 mg. In addition, the patient was using oxygen therapy and nebulizer treatment. The patient also reported taking numerous over-the-counter herbs and supplements, including 5000 IU vitamin D twice daily for a total daily dose of 10,000 IU per day, for many years. Of note, the patient’s use of vitamin D was not present in the patient’s medication list on hospital admission. At the time of presentation, the patient was self-medicating with metolazone for three days as he suspected being fluid overloaded.

On presentation, he was hemodynamically stable with mild crackles and wheezing in bilateral lung bases. Chest X-ray (CXR) showed pulmonary edema. Initial lab workup showed the patient to be hypercalcemic with acute-on-chronic kidney injury as shown in Table 1. Computed tomography (CT) abdomen/pelvis revealed bilateral non-obstructing renal calculi without hydronephrosis, as confirmed with an ultrasound of the kidneys. The workup of hypercalcemia, as shown in Table 2, identified hypervitaminosis D as the cause of the patient’s clinical presentation with elevated serum 25-OH-vitamin D and low serum parathyroid hormone-related protein (PTHrP).

**Table 1:** Laboratory values on the day of admission *Patient Baseline 1.1-1.4 mg/dL, **Chronically low due to myelodysplastic syndrome Pro-BNP: pro-b-type natriuretic peptide

Blood Test	Results
Sodium (136-145 mmol/L)	135
Potassium (3.4-5.1 mmol/L)	4.8
Chloride (98-107 mmol/L)	92
Bicarbonate (22-29 mmol/L)	26
Blood Urea Nitrogen (8-23 mmol/L)	66
Creatinine (0.70-1.20 mg/dL)	7.43*
Calcium (8.8-10.2 mg/dL)	12.8
White Blood Cells (4.5-10.5 x 103/μL)	15.6
Hemoglobin (13.2-16.6 g/dL)	10**
Platelet Count (135-317 x 103/μL)	46**
Pro-BNP	27,000

**Table 2:** Hypercalcemia work-up showing elevated 25-hydroxy vitamin D levels *Upper cutoff for our laboratory, **On two separate lab draws

Laboratory Test	Results	Hospital Day
Serum Vitamin D 25-Hydroxy (>30 ng/mL)	>100*	Day 1
Serum Calcitriol/1,25-Dihydroxy Vitamin D (19.9-79.3 pg/mL)	55.1	Day 1
Serum Parathyroid Hormone Intact (10-65 ng/mL)	11	Day 1
Serum PTHrP (<2.5 pmol/L)	<2.0**	Day 1, Day 2
Serum Calcium Ionized (1.13-1.32 mmol/L)	1.45	Day 1
Serum Protein Electrophoresis Interpretation	NORMALLY MIGRATING SERUM PROTEINS	Day 0
Urine Protein Electrophoresis Interpretation	MILD PROTEINURIA IN TUBULAR NON SELECTIVE PATTERN	Day 0
24-Hour Urine Angiotensin Converting Enzyme (U/L)	<15	Day 1

The patient was treated with intravenous normal saline and intravenous furosemide as adjusted by the nephrology team and was placed on a five-day course of 20 mg prednisone PO daily. Metolazone and all over-the-counter supplements were held. The patient’s chief complaint of leg pain waxed and waned throughout the hospital course and was still present on discharge (Day 7) without any confirmed etiology. A trend of the patient’s pertinent serum chemistry values over the hospital stay and on follow-up shows an improvement in kidney function and serum calcium levels, as shown in Table 3.

**Table 3:** Trend in laboratory values over the course of hospitalization and follow-up *Values for AM and PM blood draws, respectively

Day	Day 0	Day 2*	Day 7 (Discharge)	Day 28 (Follow-up)
Blood Urea Nitrogen (mg/dL)	66	84, 86	55	23
Creatinine (mg/dL)	7.43	8.82, 8.59	3.87	1.53
Calcium (mg/dL)	12.8	11.6, 12.0	8.1	9.9
Phosphorus (mg/dL)	7.2	6.9, 7.4	3.2	-
Magnesium (mg/dL)	2.9	2.7, 2.6	1.8	2.1

## Discussion

Vitamin D is a key regulator of calcium homeostasis of the body and is being increasingly recognized in the non-calcium-related functions of the body, notably in osteoclast proliferation via the receptor activator of the nuclear factor-kappa-Β ligand (RANKL) pathway, parathyroid gland proliferation, and in protection against many auto-immune disorders and cancers [2-3]. Vitamin D deficiency is prevalent in North America across all age groups and ethnicities, as determined by multiple studies [4-6]. Correction of deficiency with oral vitamin D supplementation of 50,000 IU/week for six to eight weeks is the standard of care. But, despite a wide therapeutic index, overzealous correction by the physicians or unsupervised consumption of supplements by the patients, is resulting in increasingly prevalent cases of VDT. A cross-sectional study in the United States from 1999 to 2014 determined that the prevalence of vitamin D supplementation ≥4000 IU/day had increased from <0.1% prior to 2005-2006 to 3.2% (2.5%-4.0%) in 2013-2014 [7]. The guidelines by the Food and Nutrition Board of the USA specify 2000I U/day as the highest vitamin D intake that healthy adults can consume without risk of hypercalcemia [8]. Our patient was taking 10,000 IU/day vitamin D for many years, in the background of chronic kidney disease, which likely predisposed him to develop AKI with a serum concentration of 25(OH)D of >100 ng/mL. 

Clinically, VDT appears similar to other hypercalcemic states, with neuropsychiatric, gastrointestinal, cardiovascular, and renal manifestations [9-10]. The treatment of acute VDT involves the discontinuation of vitamin D supplementation and reduction in dietary calcium intake. First-line treatment includes hydration with isotonic normal saline and loop diuretics, along with glucocorticoids, calcitonin, and/or bisphosphonates. Second-line agents include phenobarbital, ketoconazole, aminoquinolines, and specific inhibitors of CYP27B1 [10]. Physicians are also encouraged to inform the patients about the water-soluble and fat-soluble properties of vitamins, with special emphasis on the fat-solubility of vitamin D, which makes it more likely to accumulate in body fat and cause toxicity. Moreover, apart from the exogenous consumption of vitamin D, physicians should also be mindful of the endogenous causes of hypervitaminosis D and hypercalcemia-like granulomatous disorders, lymphomas, Williams-Beuren syndrome, and idiopathic infantile hypercalcemia, to name a few, and order the pertinent work-up wherever necessary.

## Conclusions

The seemingly innocuous consumption of vitamin D, in large amounts, can result in the development of vitamin D toxicity. It is important to increase awareness about the correct use of vitamin D among patients, as well as physicians and other health care providers. It is recommended that primary care providers have conversations with their patients about the use of over-the-counter vitamins and supplements, and counsel them in regards to the potentially deleterious effects of the misinformed use of these products.
